# Automatic Segmentation and Evaluation of Mitral Regurgitation Using Doppler Echocardiographic Images

**DOI:** 10.3390/bioengineering11111131

**Published:** 2024-11-09

**Authors:** Guorong Liu, Yulong Wang, Hanlin Cheng, Zhongqing Shi, Zhanru Qi, Jing Yao, Shouhua Luo, Gong Chen

**Affiliations:** 1 School of Artificial Intelligence and Information Technology, Nanjing University of Chinese Medicine, Nanjing 210023, China; 20221138@njucm.edu.cn (G.L.); 20231069@njucm.edu.cn (Y.W.); 2 School of Biological Science and Medical Engineering, Southeast University, Nanjing 211102, China; chenghanlin@seu.edu.cn (H.C.); luoshouhua@seu.edu.cn (S.L.); 3 The Affiliated Hospital of Nanjing University Medical School, Nanjing Drum Tower Hospital, Nanjing 210008, China; 13260986075@126.com (Z.S.); zhanruqi7@163.com (Z.Q.); jingyao@nju.edu.cn (J.Y.); 4 Affiliated Hospital of Nanjing University of Traditional Chinese Medicine, Nanjing 210029, China

**Keywords:** mitral regurgitation, deep learning, semantic segmentation

## Abstract

Background: Mitral Regurgitation (MR) is a common heart valve disease. Severe MR can lead to pulmonary hypertension, cardiac arrhythmia, and even death. Therefore, early diagnosis and assessment of MR severity are crucial. In this study, we propose a deep learning-based method for segmenting MR regions, aiming to improve the efficiency of MR severity classification and diagnosis. Methods: We enhanced the Efficient Multi-Scale Attention (EMA) module to capture multi-scale features more effectively, thereby improving its segmentation performance on MR regions, which vary widely in size. A total of 367 color Doppler echocardiography images were acquired, with 293 images used for model training and 74 images for testing. To fully validate the capability of the improved EMA module, we use ResUNet as the backbone, partially integrating the enhanced EMA module into the decoder’s upsampling process. The proposed model is then compared with classic models like Deeplabv3+ and PSPNet, as well as UNet, ResUNet, ResUNet with the original EMA module added, and UNet with the improved EMA module added. Results: The experimental results demonstrate that the model proposed in this study achieved the best performance for the segmentation of the MR region on the test dataset: Jaccard (84.37%), MPA (92.39%), Recall (90.91%), and Precision (91.9%). In addition, the classification of MR severity based on the segmentation mask generated by our proposed model also achieved acceptable performance: Accuracy (95.27%), Precision (88.52%), Recall (91.13%), and F1-score (90.30%). Conclusion: The model proposed in this study achieved accurate segmentation of MR regions, and based on its segmentation mask, automatic and accurate assessment of MR severity can be realized, potentially assisting radiologists and cardiologists in making decisions about MR.

## 1. Introduction

Mitral regurgitation (MR) is a prevalent heart valve disease in clinical practice [1], with its incidence increasing with age, reaching up to 10% in individuals over 75 years old [2]. Without therapeutic intervention, severe MR can lead to complications such as cardiac arrhythmias and significantly elevate the risk of mortality [1]. Additionally, the distribution of MR severity is uneven, with mild MR being relatively more prevalent. A large-scale echocardiography database analysis conducted by a team from Zhongshan Hospital affiliated with Fudan University in 2016 revealed detection rates of mild, moderate, and severe MR at 42.44%, 1.63%, and 1.44%, respectively [3]. Similarly, a large-sample survey conducted by a team from the Second Affiliated Hospital of Zhejiang University School of Medicine in 2017 reported a detection rate of 0.68% for severe MR [4]. Hence, an accurate assessment of MR severity is essential for preoperative assessment, intraoperative monitoring, and postoperative evaluation of MR patients.

Transthoracic echocardiography (TTE) is the most commonly used imaging technique for the diagnostic evaluation of MR due to its safety, non-invasiveness, and efficiency [5]. It plays a crucial role throughout the clinical diagnosis and treatment of MR, as it not only allows for direct visualization of the morphology and extent of MR but serves as a reference for determining the need for surgical intervention by monitoring MR progression [6]. Currently, the assessment of MR primarily relies on the subjective judgment of clinicians based on their clinical experience, leading to issues such as high labor costs, poor reproducibility, and inconsistency. In this context, image segmentation techniques offer promising applications for the automatic and precise delineation of regurgitation regions. Effective segmentation can enhance the accuracy of ultrasound image analysis, facilitate the identification and localization of lesion areas, and ultimately improve the early detection and treatment of MR [7].

In recent years, artificial intelligence (AI) has been increasingly utilized in ultrasound medicine, particularly in the field of cardiovascular diseases. Tim et al. [8] developed a novel machine learning (ML) risk stratification tool for predicting the 5-year risk in hypertrophic cardiomyopathy (HCM). Ahmed S. Fahmy et al. [9] proposed an ML-based model to identify individual HCM patients at high risk of developing advanced heart failure symptoms. Tae-Min Rhee et al. [10] established ML-based models to discriminate major cardiovascular events in patients with HCM. However, there is limited research on the use of AI for the assessment of MR severity, particularly from TTE, which mainly focuses on two aspects: the classification of MR severity and the segmentation of the MR region. Balodi et al. [11] utilized texture feature extraction with a multiresolution local binary pattern (MLBP) variant for the classification of MR severity and achieved high classification accuracy. Penso et al. [12] explored the application of ML methods in predicting MR recurrence. However, the association between echocardiography and surgical variables with MR recurrence remains controversial, and the potential causes are not yet well understood. Regarding the segmentation of the MR region, Atika et al. [13] conducted a preliminary study comparing the performance of multiple deep learning (DL) image segmentation algorithms for MR region segmentation, finding that U-Net3 was superior in performance. U-Net3 [14] is a variant of U-Net architecture, but it is difficult to be applied to clinical application scenarios due to the high computational complexity and long inference time. Huang et al. [15] proposed a deep learning (DL)-based automated MR and tricuspid regurgitation (TR) segmentation and classification method, VABC-UNet. Although the segmentation accuracy reached 0.85 for the TR region, it was only 0.7 for the MR region. The morphology of MR in medical images is highly variable and exhibits irregularity, this model may produce inaccurate segmentation when dealing with complex cases. Yang et al. [16] also proposed a self-supervised learning algorithm for MR region segmentation, indicating that the accuracy of MR assessment still requires improvement. Moreover, there are end-to-end MR severity assessment studies. Zhang et al. [17] attempted to use Mask Region-Based Convolutional Neural Network (Mask R-CNN) for automatic qualitative assessment of MR, finding that the assessment accuracy for moderate MR samples was only 0.81. This was primarily due to the partial overlap between the features of grade III (moderate) MR and grade IV (severe) MR, making it difficult to distinguish between moderate and severe MR. Long et al. [18] proposed a DL system named DELINEATE-MR, which processes complete TTE videos to classify MR; however, the spatiotemporal convolution used in this model struggles with small-scale irregular targets, leading to inaccurate segmentation of MR region boundaries.

UNet [19] is a convolutional neural network (CNN) architecture widely accepted in the field as a classical medical image segmentation model due to its high accuracy and effectiveness with small samples. Although UNet and its variants, such as UNet3 [14], have been shown to segment the MR region efficiently, their segmentation accuracy still requires improvement due to the irregularities present in the MR region. ResNet [20] is well-known in the field of DL for addressing issues such as gradient vanishing through the introduction of residual blocks, and it also demonstrates excellent performance when combined with UNet [21]. By combining ResNet and UNet, it is possible to design a highly effective method for segmenting the MR region in TTE, which can subsequently be utilized for MR severity classification.

Based on this, in this study, we used UNet as the backbone and selected ResNet50 as the encoder. We then improved the Efficient Multi-Scale Attention (EMA) module for MR region segmentation, aiming to effectively capture multi-scale features to enhance segmentation accuracy, particularly for small-scale irregular targets. To further validate the effectiveness of the improved EMA module, we integrated it into the upsampling part of the UNet decoder.

## 2. Materials and Methods

**Figure 1:**
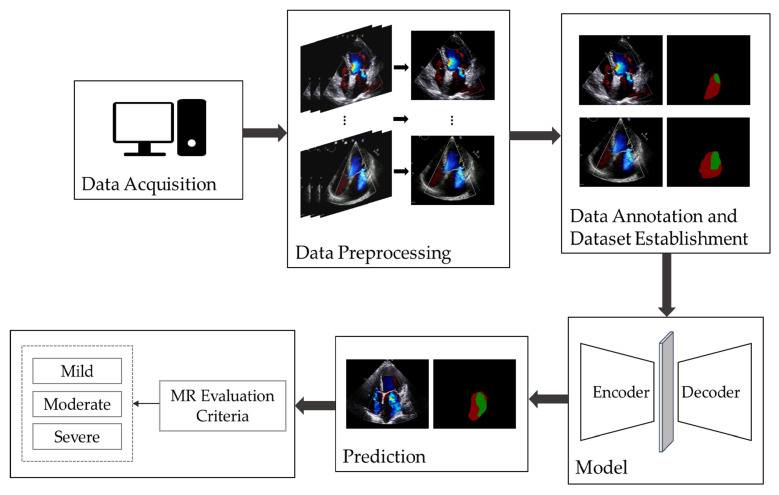
The overall workflow diagram of automatic MR segmentation and evaluation. The images are apical four-chamber views, the green pixel block is MR region and the red pixel block is LA region in the annotated data. MR: Mitral Regurgitation; LA: left atrium.

Figure 1 illustrates the overall workflow of automatic MR segmentation and evaluation in this study. We began by selecting apical four-chamber view color Doppler echocardiographic videos and extracted the frames displaying maximal regurgitation. Following this, we performed data annotation and constructed the dataset and proposed an improved UNet model for the automatic segmentation and evaluation of MR.

### 2.1. Establishment of Dataset

Since there is no publicly available dataset for MR echocardiography, this study was conducted using a private dataset constructed by our team. The dataset comprises 367 MR apical four-chamber view color Doppler echocardiographic images, with 100, 137, and 130 images categorized as having mild, moderate, and severe MR severity, respectively. All images were labeled and preprocessed accordingly.

#### 2.1.1. Data Acquisition and Cleaning

This study utilized the ultrasound information system of Gulou Hospital, affiliated with Nanjing University School of Medicine, to select a total of 367 images from apical four-chamber color Doppler echocardiograms labeled with “mitral regurgitation”. These images were acquired by the Department of Echocardiography from January 2023 to October 2023. The dataset includes 100 images with mild MR, 137 with moderate MR, and 130 with severe MR. During the data acquisition process, low-quality and unclear images, as well as data from cases of easily overestimated eccentric MR, were excluded [22,23]. Statistical analysis revealed that 56.95% of the patients included in this study were female, and 73.56% were over 50 years of age. Additionally, the average age of the patients tended to increase with the severity of MR. The images were obtained using Philips Medical Systems and GE Vingmed Ultrasound devices, and they were saved in DICOM format.

#### 2.1.2. Data Preprocessing and Annotation

**Figure 2:**
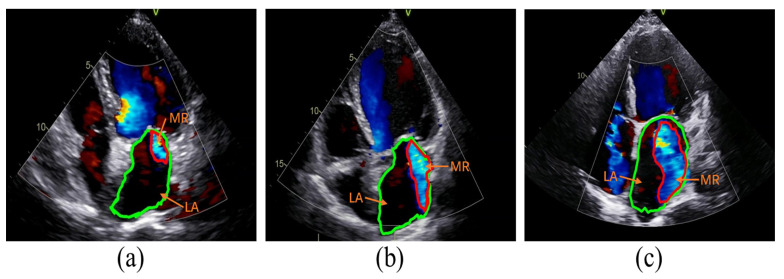
Examples of MR Severity: (**a**–**c**) show mild (RF < 30), moderate (30 < RF < 50), and severe (RF > 50) MR, respectively. The images are apical four-chamber views, with the green contour marking the LA border and the red contour marking the MR border. MR: Mitral Regurgitation; RF: Regurgitation Fraction; LA: Left Atrium.

The classification of MR severity was based on the criteria outlined in the American Society of Echocardiography guidelines [6]. Guideline references for assessing the severity of MR include vena contracta width (VCW), effective regurgitant orifice area (EROA), regurgitant volume (RVol), and regurgitation fraction (RF). To avoid discrepancies in the size of images acquired by different devices, RF was chosen as a semi-quantitative index of MR severity in this study. Specifically, the ratio of the area of MR to the area of the left atrium (LA) was used as RF. A fraction of less than 30% corresponds to mild MR, 30–49% corresponds to moderate MR, and greater than or equal to 50% corresponds to severe MR, as illustrated in Figure 2.

**Figure 3:**
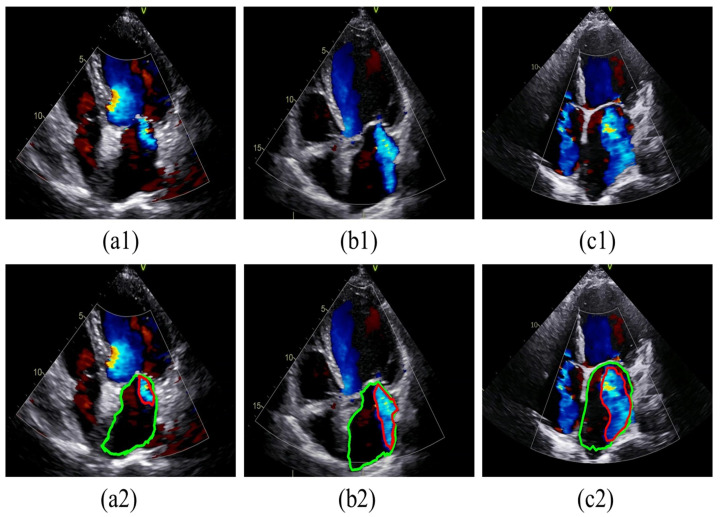
Examples of MR labeled. (**a1**–**c1**) show original images of mild, moderate, and severe MR, respectively. (**a2**–**c2**) show the labeled images of MR corresponding to (**a1**–**c1**) sequentially. Red contour: MR; Green contour: LA. MR: mitral regurgitation; LA: left atrium.

To facilitate presentation and annotation, the collected color Doppler echocardiograms were converted from DICOM format to MP4 format. Frames that most accurately represented the regurgitation were selected from each video. Subsequently, all images were standardized by defining sectors as regions of interest (ROI) and then resized to 800 × 600 pixels after cropping. The MR regions and LA boundaries in all relevant frames were annotated by two specialized physicians using Labelme software (version 5.3.1). In cases of disagreement, a third physician was consulted for final judgment, as shown in Figure 3. The labeled images were then divided into training and test sets at a ratio of 8:2. Specifically, 293 images were allocated to the training dataset, while 74 images were designated for the testing dataset, as detailed in Table 1.

### 2.2. Establishment of the Model for Segmentation

**Figure 4:**
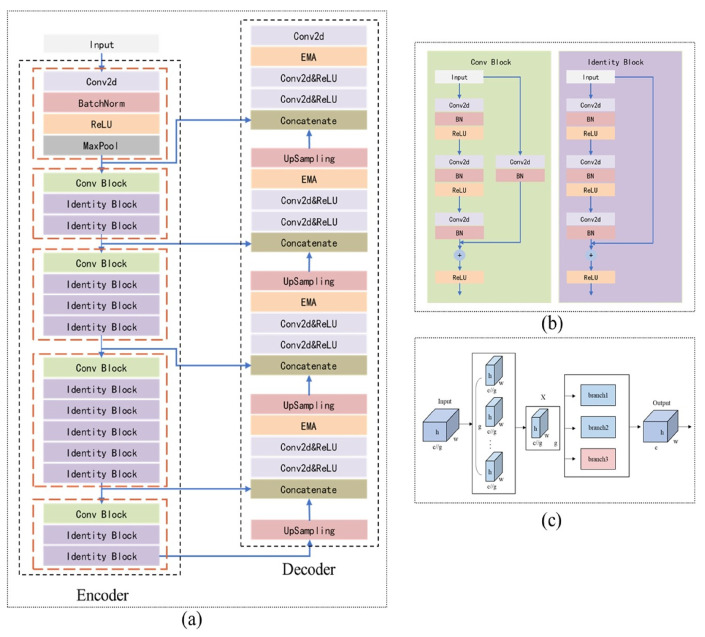
The model architecture proposed in this study. (**a**) The overall architecture of the improved UNet model. The orange-red dashed box in the Encoder highlights the correspondence to stages 0 through 4 of ResNet, from top to bottom. (**b**) The structure of the Conv Block and Identity Block within the improved UNet architecture. (**c**) The structure of the enhanced EMA module, with the light-red areas indicating modifications made to the original EMA module in this study.

In this study, we utilize ResUNet as the backbone, incorporating ResNet as the encoder in UNet, and introducing an improved Efficient Multi-Scale Attention (EMA) module during the upsampling stage of the decoder, as depicted in Figure 4.

#### 2.2.1. Model Building

The model proposed in this study is inspired by both the UNet and ResNet architectures. UNet [19] is a CNN designed for image segmentation, characterized by its “U” shape, which consists of an encoder and a decoder. The encoder extracts image features, while the decoder recovers spatial resolution. During the UNet segmentation process, spatial information is compressed and subsequently recovered. The decoder’s upsampling layer employs 2 × 2 deconvolution to double the size of the feature maps, which are then integrated with the corresponding encoder outputs through skip connections, preserving essential details. However, this method does not completely prevent the loss of fine spatial information during the encoder’s downsampling phase, especially when the encoder has limited feature extraction capabilities.

ResNet [20] is a simple yet effective CNN architecture known for its strong feature extraction capabilities. ResNet introduces a residual unit that simplifies the learning process by adding a shortcut connection, preserving the original output of the previous layer and transforming the network’s learning task into the learning of the residual function F(x) = H(x) − x. By addressing the issues of gradient vanishing and exploding in deep networks through residual learning, ResNet enables the effective training of networks with hundreds or even thousands of layers, thus extending the model’s potential performance limits.

ResNetU-Net [21] is a variation of the U-Net architecture that replaces the “convolution-normalization-activation” layers with residual units to construct its basic blocks. The benefits of this approach include: (1) residual units simplify network training; and (2) internal skip connections and information propagation enhance design simplicity and performance. However, because the residual unit is based on convolution operations, which are fundamentally local filters, it can only capture relationships between local features. This limitation restricts the ability to model spatial information for multi-scale and irregularly shaped MR regions, potentially leading to inadequate restoration of spatial resolution during the upsampling stage.

To address these challenges, this study improves the UNet model by integrating the initial stages of ResNet50 into the UNet encoder-decoder structure, using it as the encoder (Figure 4a,b). This enhancement boosts the model’s feature extraction capabilities and preserves as much spatial information as possible during downsampling. Additionally, an improved EMA module (Figure 4c) is added to the decoder to enhance the model’s ability to recover spatial information.

#### 2.2.2. Improved EMA Module

The Channel Attention Mechanism (CAM) [24] enhances feature map representation by assigning higher weights to important channels. However, reducing channel dimensions to model cross-channel relationships may limit the extraction of deep visual representations.

The Efficient Multi-Scale Attention (EMA) module [25] retains the information of each channel while reducing computational overhead. It reshapes partial channels into batch dimensions and groups channel dimensions into multiple sub-features, ensuring well-distributed spatial semantic features within each feature group. The EMA module recalibrates channel weights in each parallel branch by encoding global information and captures pixel-level pairwise relationships through cross-dimensional interactions. It employs two convolutional kernels in separate parallel subnets: a 1 × 1 branch that models cross-channel information using global average pooling and a 3 × 3 branch that aggregates multi-scale spatial structure information.

**Figure 5:**
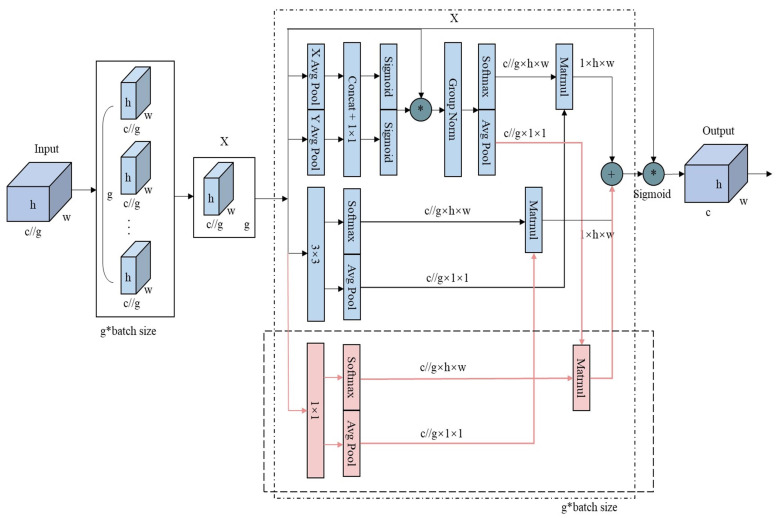
The improved EMA module architecture. Blue blocks: the original EMA architecture, Light-red blocks: the addition of 1 × 1 parallel branch for module fusion.

The original EMA module uses two parallel branches with relatively simple feature extraction methods: a 1 × 1 global pooling branch for global information and a 3 × 3 branch for local information. However, this setup may be insufficient for segmentation tasks involving multi-scale or small-scale irregular targets, as it struggles to balance weight and information complementation, limiting the richness of feature expression. To overcome this limitation and enhance the model’s ability to capture multi-scale information and complex patterns, this study introduces an additional 1 × 1 parallel branch. This new branch facilitates a more comprehensive integration of global and local information. As shown in Figure 5, the average pooling (avgpool) output from the original 1 × 1 branch is separated from the softmax output of the 3 × 3 branch. The avgpool output from the original 1 × 1 branch is then multiplied by the softmax output of the newly added 1 × 1 branch, while the avgpool output of the newly added 1 × 1 branch is multiplied by the softmax output of the 3 × 3 branch. These results are summed with the outputs from the original branches, effectively integrating the new 1 × 1 branch with the original EMA module’s branches. Finally, the outputs from the three branches are adjusted using sigmoid activation and normalization to produce the final feature map.

#### 2.2.3. Loss Function

The loss function employed in this study combines focal loss and Dice loss, as shown in Equation (1).

Focal loss [26] is an improved cross-entropy loss method designed to address the category imbalance problem between the target object and the background class in semantic segmentation, helping to mitigate the impact of this imbalance. The calculation of focal loss is provided in Equation (2), where $p_{t}$ represents the predicted probability, $\alpha_{t}$ is a weight factor that balances positive and negative samples, and γ is the focusing parameter that adjusts the weight distribution between easy and difficult samples.

Dice loss [27] is derived from the Dice coefficient, designed to evaluate the similarity between segmentation results and ground truth labels. It directly optimizes the overlap between the predicted segmentation and the true labels, thereby improving segmentation accuracy, particularly for small-scale targets. The Dice loss calculation is presented in Equation (3), where $p_{i}$ and $g_{i}$ represent the predicted value and the ground truth label at the i-th pixel, respectively.

(1) $$L=L_{focal}+L_{Dice}$$

(2) $$L_{focal}=-\alpha_{t}{\left(1-p_{t}\right)}^{\gamma }log(p_{t})$$

(3) $$L_{Dice}=1-\frac{2\sum_{i=1}^{N}p_{i}g_{i}}{\sum_{i=1}^{N}p_{i}^{2}+\sum_{i=1}^{N}g_{i}^{2}}$$

### 2.3. Implementation Details

The experiments were conducted on a system with an Intel(R) Core(TM) i5-10500 processor and a single GeForce RTX3060 GPU with 12 GB of video memory. The code was implemented in an environment configured with Windows 10, CUDA 11.1.3, cuDNN 8.0, and PyTorch (v1.9.0, Python 3.8). Given the limited dataset size, data augmentations such as random rotation and random scaling were applied.

The model was trained for 100 epochs using the Adam optimizer. The initial learning rate was set to 0.0001, with beta values of (0.9, 0.999) and a weight decay of 0. The batch size was set to 2. During the first 50 epochs, the initial weights of ResNet50, pre-trained on the IMAGENET1K_V2 dataset, were used, and the weights of the encoder were frozen to stabilize training.

### 2.4. Evaluation Metrics and Comparison Methods

In this study, the proposed model was compared with several classical segmentation models, including DeepLabv3+, PSPNet, UNet, ResUNet, ResUNet with the original EMA, and UNet with the improved EMA. DeepLabv3+ [28] employs an encoder-decoder architecture, where the encoder stage leverages atrous convolution to capture multi-scale contextual information, while the decoder stage refines segmentation results, particularly at object boundaries. PSPNet [29] achieves semantic segmentation through a pyramidal scene parsing network, integrating global contextual information across different regions to enhance prediction accuracy and reliability. Both of these models are extensively used in medical image segmentation and demonstrate notable performance advantages.

In the experiment, four evaluation metrics—Jaccard, Mean Pixel Accuracy (MPA), Precision, and Recall—are employed to quantitatively assess the model’s performance in segmenting regurgitation and atria. Additionally, Precision, Recall, Accuracy, and F1-score were used to evaluate the model’s classification performance. The formulas for these metrics are provided in Equations (4)–(9), where TP represents the number of true positives, TN denotes the number of true negatives, FP indicates the number of false positives, and FN stands for the number of false negatives. Detailed definitions of the metrics are included in the Supplementary Materials.

(4) $$Jaccard=\frac{TP}{TP+FP+FN}$$

(5) $$Precision=\frac{TP}{TP+FP}$$

(6) $$Recall=\frac{TP}{TP+FN}$$

(7) $$MPA=\frac{1}{N}\sum_{i=1}^{N}{Accuracy}_{i}$$

(8) $$Accuracy=\frac{TP+TN}{TP+TN+FP+FN}$$

(9) $$F1-score=\frac{2TP}{2TP+FN+FP}$$

## 3. Results

### 3.1. Comparison of Segmentation Results

Table 2 presents the quantitative results of the improved ResUNet model compared to other models, including DeepLabv3+, PSPNet, UNet, ResUNet, ResUNet with the original EMA, and UNet with the improved EMA. The improved ResUNet model achieves a Jaccard score of 84.37%, a MPA of 92.39%, a Precision of 91.9%, and a Recall of 90.91% in the MR region segmentation task. This model outperforms all others in both Jaccard and MPA metrics, demonstrating strong overall performance. This indicates that our proposed model achieves higher accuracy and consistency in identifying and segmenting MR regions. Specifically, ResUNet combined with the improved EMA outperforms ResUNet with the original EMA in Jaccard, MPA, and Precision. Although the improved EMA slightly reduces Recall compared to the original EMA (by 0.07%), the difference is minimal. UNet shows poorer performance than ResUNet with EMA in Jaccard, MPA, and Recall metrics. However, when combined with the improved EMA module, UNet surpasses ResUNet with the original EMA in Jaccard and Precision. These results highlight the effectiveness of the improved EMA module proposed in this study, demonstrating its superiority in enhancing model performance.

**Figure 6:**
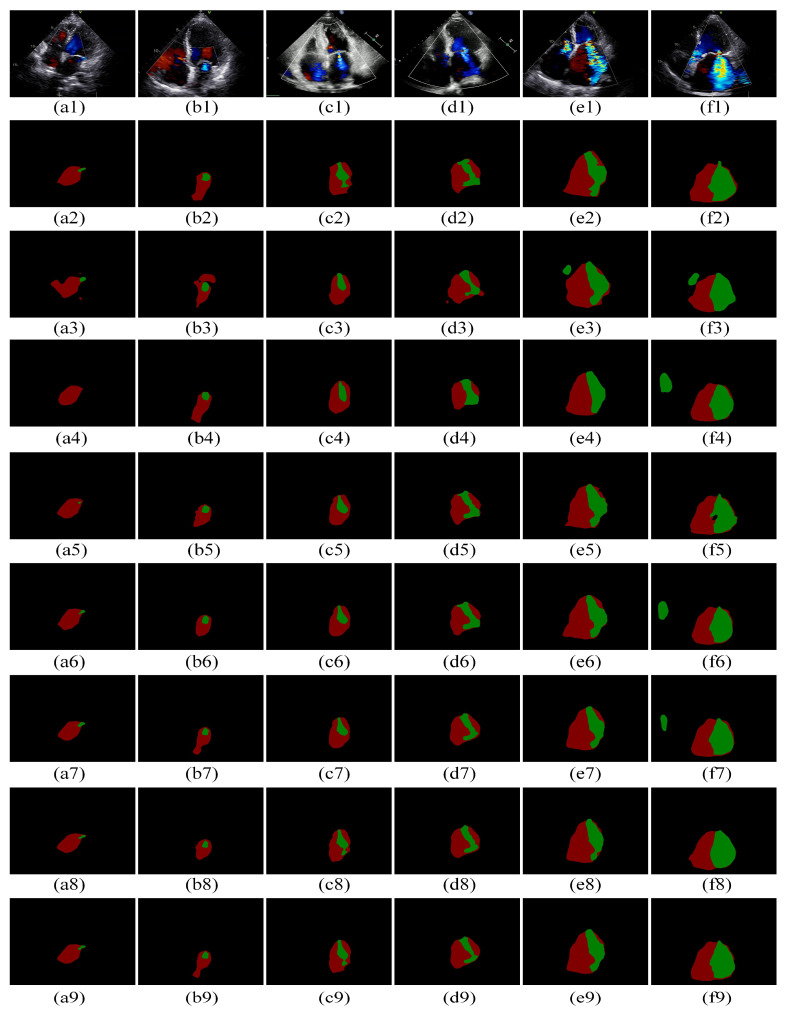
Comparison of our model with the other models. (**a1**–**a9**): MR images, ground truth, the prediction plots of Deeplabv3+, PSPNet, UNet, ResUNet, ResUNet + EMA, UNet + improved EMA, ResUNet + improved EMA, and ResUNet + improved EMA, respectively; (**b1**–**f9**) are the different MR images corresponding to (**a1**–**a9**), respectively. The green pixel blocks are MR regions and the brown-red pixel blocks are LA regions. MR: mitral regurgitation; LA: left Atrium.

Figure 6 shows the segmentation masks of the proposed ResUNet with the improved EMA compared to those of DeepLabv3+, PSPNet, UNet, ResUNet, ResUNet with the original EMA, and UNet with the improved EMA. Figure 6 illustrates that ResUNet provides more accurate recognition of MR and LA contours with less over-segmentation compared to DeepLabv3+ and PSPNet (Figure 6(f2–f5)). Over-segmentation occurs when the model delineates the regurgitant area with excessive detail, misclassifying surrounding normal tissue as part of the lesion, thereby compromising the accurate assessment of the pathology. ResUNet with the improved EMA performs better than ResUNet with the original EMA, particularly in challenging cases such as MR regions with subtle color variations, where it segments MR edges more precisely (Figure 6(c1,c2,c7,c9)). The improved EMA module also enhances the performance of UNet relative to the original EMA, resulting in fewer pseudo-segmentations (Figure 6(f2,f6,f8)). Pseudo-segmentation refers to the model mistaking normal tissue for regurgitation, which can lead to clinical misdiagnosis or overtreatment. Additionally, ResUNet with the improved EMA accurately recognizes irregular MR and LA regions (Figure 6(b2,b8,b9)). These results indicate that ResUNet with the improved EMA model reduces over-segmentation compared to models without EMA and provides superior segmentation of MR contours compared to models with EMA added to UNet, demonstrating the effectiveness of the proposed model in segmenting MR and LA regions.

### 3.2. Qualitative Effects of Loss Function

Since identical or similar loss values across different loss functions cannot be directly compared, this study primarily assesses the impact of various loss functions on model performance through a qualitative comparison of the segmentation masks. This comparison involves examining the profiles of segmentation masks generated by models trained with different loss functions.

**Figure 7:**
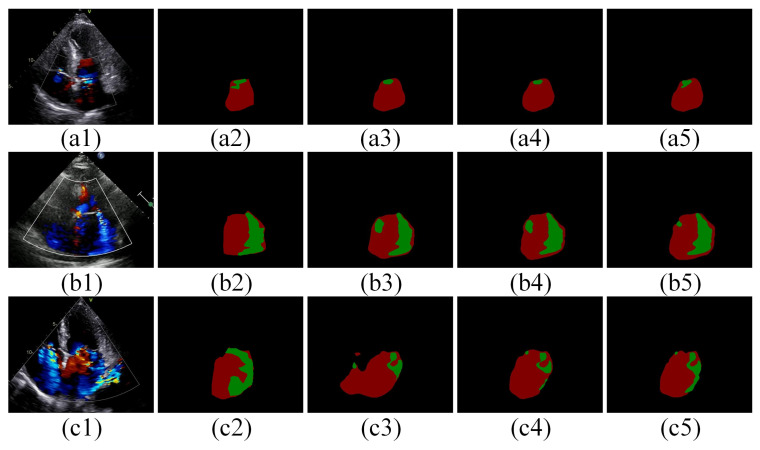
The effect of loss on the results. (**a1**–**a5**) are the original mild MR image, ground truth, and the predicted images using focal loss, dice loss, and focal loss + dice loss, respectively; (**b1**–**b5**) and (**c1**–**c5**) are images of the moderate MR image and the severe MR image corresponding to (**a1**–**a5**), respectively. The green pixel blocks are MR regions, and the brown-red pixel blocks are LA regions. MR: mitral regurgitation; LA: left atrium.

Figure 7 demonstrates the visual comparison of segmentation results from the models proposed in this study after training with different loss functions. The models trained using the combination of Focal loss and Dice loss (Equation (1)) (Figure 7(a5,b5,c5)) exhibit fewer pseudo-segmentations and over-segmentations compared to models trained with only focal loss (Equation (2)) (Figure 7(a3,b3,c3)). The edges of the segmented masks align more closely with the ground truth, particularly in the MR region, than those from models trained solely with Dice loss (Equation (3)) (Figure 7(a4,b4,c4)).

In this study, the combination of Focal loss and Dice loss enhances the model’s ability to focus on the overall shape and boundary of the MR region, which is characterized by its small scale and irregularity, as well as the significant difference between the MR region and the left atrial area.

### 3.3. MR Automated Assessment Result

**Figure 8:**
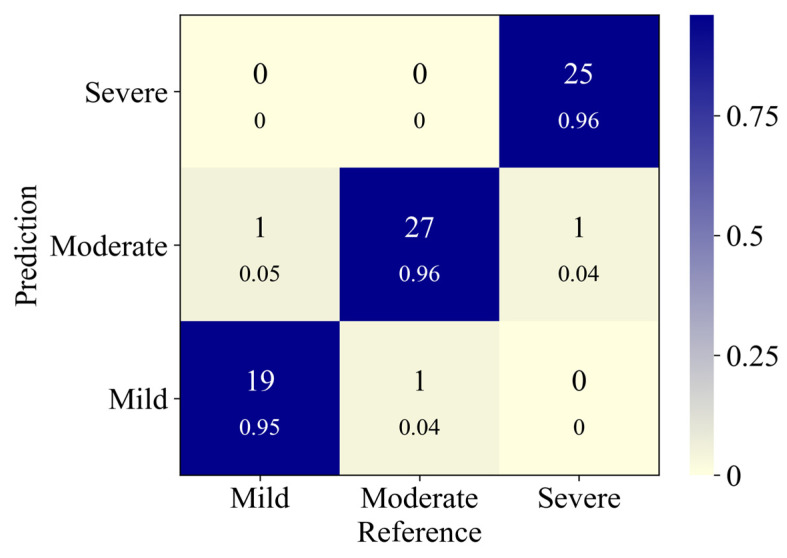
Confusion matrix for the model’s classification of MR severity. MR: mitral regurgitation.

Based on the segmentation masks generated by the model proposed in this study, MR severity was assessed for samples with varying degrees of regurgitation severity in the test dataset, as shown in Table 3. The classification of MR achieved an accuracy of 95.27%, a precision of 88.52%, a recall of 91.13%, and an F1-score of 90.30%. Notably, the accuracy for the mild, moderate, and severe MR categories reached approximately 95%. The confusion matrix (Figure 8) illustrates the classification performance across different severities of MR. The diagonal elements represent the correct classification rate for each category, with the number of correctly classified samples displayed above the accuracy rate. In contrast, the off-diagonal elements highlight instances of misclassification, particularly between mild and moderate MR, as well as between moderate and severe MR. This misclassification primarily arises from the imaging similarities between certain cases of mild and moderate MR and between moderate and severe MR.

## 4. Discussion

Mitral Regurgitation (MR) is a common and serious heart valve disease that significantly impacts patient prognosis. Accurate diagnosis and effective treatment are crucial for improving outcomes. Therefore, developing automated tools to enhance the accuracy and consistency of MR diagnosis is vital for evaluating the disorder, formulating treatment strategies, and conducting postoperative surveillance. Previous research on MR region segmentation using DL methods has faced challenges such as suboptimal accuracy [13,15], significant variability in category-specific accuracy [17], and imprecise segmentation boundaries [18], which complicate meeting clinical requirements. UNet is widely recognized as a classical model for medical image segmentation, and its variants have demonstrated excellent performance in valve regurgitation segmentation [13,15]. However, these models still have some limitations. In this study, we proposed an advanced UNet-based segmentation model designed to assess MR severity more accurately. This model utilizes ResNet as the encoder within the UNet architecture, enhancing feature extraction during the downsampling process to preserve maximal spatial information. Additionally, an improved EMA module is integrated during the upsampling phase to capture multi-scale data more effectively and improve the reconstruction of fine spatial details in anatomical structures.

Our improved UNet model was benchmarked against several prominent segmentation methods, including Deeplabv3+, PSPNet, UNet, ResUNet, and ResUNet with the EMA module, for the MR segmentation task. The experimental results demonstrate that our model significantly outperforms these methods, particularly in the Jaccard (84.37%) and MPA (92.39%) metrics, achieving top-tier performance in Precision (91.9%) and Recall (90.91%), leading the benchmarks in the former two metrics (Table 2). Moreover, ResUNet augmented with the improved EMA module surpasses the original configuration across Jaccard, MPA, and Precision metrics (Table 2). This improvement underscores the enhanced efficacy of the improved EMA module within the UNet framework, particularly noted in the Jaccard index against ResUNet with the EMA module (Table 2). These findings highlight the advanced capability of the improved EMA module in MR segmentation tasks. In terms of MR severity classification, the segmentation masks derived from our model achieved an overall accuracy of 95.27%, with Precision at 88.52%, Recall at 91.13%, and an F1 score of 90.30%. The model accurately classifies mild, moderate, and severe MR stages with respective accuracies of 94%, 95%, and 96% (Table 3), demonstrating its high precision. Qualitative analysis of the segmentation results from different models shows that our proposed model is more accurate in segmenting contour edges with less over-segmentation (Figure 6). This suggests that diversifying the spatial information extraction by increasing the number of branches, each focusing on a different spatial semantic distribution, helps capture spatial relationships in the image more uniformly and meticulously. This enhances the spatial representation of the features and better adapts to the specific needs and characteristics of the date in this study. Furthermore, the combination of Focal Loss and Dice Loss enhances the model’s ability to handle category-imbalanced pixels while capturing finer edge details of the target (Figure 7).

The quantitative results of segmentation and MR severity categorization in this study may differ significantly from those reported in other related studies. For instance, our model achieved a Jaccard score of 0.84, which is notably higher than the 0.70 reported by Huang et al. [13]. This disparity can largely be attributed to differences in the size of the test sets and sample distributions between the studies. Huang et al. [13] included both MR and TR data, whereas our study focused exclusively on MR. Moreover, we selected only the most representative frames from each echocardiographic sample to ensure high data quality. In terms of MR severity classification, the segmentation mask of our proposed model demonstrated superior results. This enhancement is likely due to the different classification criteria adopted in our study, which follows guidelines [6] categorizing MR severity into mild, moderate, and severe. In contrast, studies like Zhang et al. [17] employ a more granular classification: grade I (mild), grade II (mild–moderate), grade III (moderate), and grade IV (severe). The high degree of imaging similarity between grade III and grade IV in Zhang’s study posed challenges in accurately distinguishing between moderate and severe MR cases.

During this study, we encountered several challenges. A primary issue was the difficulty in acquiring moderate and severe MR data labeled by physicians in real clinical settings. Although data augmentation strategies partially mitigated the impact of data scarcity on model training; a more extensive and comprehensive evaluation using a larger dataset is essential for robust assessments. Additionally, in the data preprocessing stage, we manually selected the best frames from a single apical four-chamber view to assist model training, this step could be integrated into the model training process in future work. We also recommend incorporating multiple frames and views to enhance the comprehensiveness of the study. Overall, while the DL model demonstrated commendable performance in evaluating MR severity, metrics such as precision still require improvement, indicating a need for better handling of category imbalance in MR severity. Furthermore, this study utilized data from only two brands of ultrasound machines, which limits generalizability. To address these limitations, we plan to expand the dataset size and improve balance by incorporating more patient data across different MR severity levels. Future research will also consider factors such as left atrial size and individual chest wall structures, particularly focusing on eccentric MR, which is often overestimated. Finally, we aim to introduce multicenter studies to thoroughly assess the model’s generalization ability and segmentation accuracy, ultimately providing physicians with a more reliable tool for MR diagnosis.

## 5. Conclusions

In this study, we propose a DL-based method for segmenting MR regions to assist in the classification of MR severity, aiming to improving the efficiency of MR assessment. The experimental results indicate that the proposed method shows promise in segmenting MR regions and assessing MR severity, which may provide valuable support to physicians in their evaluations. Furthermore, this study represents an initial exploration into the potential application of DL in the field of heart valve diseases.

## Figures and Tables

**Table 1:** Dataset division in the study.

	Mild	Moderate	Severe	Total
Train	80	109	104	293
Test	20	28	26	74
Total	100	137	130	367

**Table 2:** Performance of each model on the test dataset.

Models	Evaluation Metrics(%)			
	Jaccard	MPA	Recall	Precision
Deeplabv3+	76.45	82.15	82.15	91.04
PSPNet	80.66	88.26	88.26	89.17
UNet	80.65	91.17	91.23	91.75
ResUNet	80.06	91.70	91.87	90.51
ResUNet + EMA	81.04	92.01	91.97	90.60
UNet + improved EMA	81.77	91.97	91.74	91.39
ResUNet + improved EMA	84.37	92.39	91.90	90.91

**Table 3:** Classification results of MR type.

Method	Numbers of Images	Severity	Evaluation Metrics			
			Precision	Recall	Accuracy	F1-Score
ResUNet + improved-EMA	20	Mild	87.86	89.03	94.55	88.44
	28	Moderate	88.44	91.04	95.57	89.72
	26	Severe	89.26	93.33	95.69	91.25
	74	Total	88.52	91.13	95.27	90.30

## Data Availability

Currently, the datasets generated and analyzed during the current study cannot be made publicly accessible due to privacy protection.

## Supplementary Materials

The following supporting information can be downloaded at: https://www.mdpi.com/article/10.3390/bioengineering11111131/s1. Table S1: The definition of metrics in this study. TP represents the number of true positives, TN denotes the number of true negatives, FP indicates the number of false positives, and FN stands for the number of false negatives [30,31,32].
