# Supplementary material for: Automatic Segmentation and Evaluation of Mitral Regurgitation Using Doppler Echocardiographic Images
Source: Bioengineering (Basel). 2024 Nov 9;11(11):1131. doi: 10.3390/bioengineering11111131 (PMC11591529; doi:10.3390/bioengineering11111131)
Supplement: Supplementary file 1 [file bioengineering-11-01131-s001.zip › bioengineering-3242432-supplementary.pdf]

Metrics	Definition	Equation	Reference
Accuracy	the ratio of the number of pixels predicted correctly by the model to the total number of pixels	$Accuracy = \frac{TP + TN}{TP + TN + FP + FN}$	[30]
Precision	a measure of how many pixels the model predicts are correct for a particular category	$Precision = \frac{TP}{TP + FP}$	[30]
Recall	a measure of a model's ability to correctly identify all instances of a category	$Recall = \frac{TP}{TP + FN}$	[30]
Mean Pixel Accuracy(MPA)	the average of the accuracy calculated for each class	$MPA = \frac{1}{N}\sum_{i=1}^{N} Accuracy_i$	[31]
Jaccard	the ratio of the intersection of the predicted and ground truth sets to their union, measures the similarity between two sets	$Jaccard = \frac{TP}{TP + FP + FN}$	[32]
F1-score	The harmonic mean of precision and recall	$F1 - score = \frac{2TP}{2TP + FN + FP}$	[30]

Table: the definition of metrics in this study. TP represents the number of true positives, TN denotes the number of true negatives, FP indicates the number of false positives, and FN stands for the number of false negatives.

You can find references during manuscript.
